# COVID-19 Infection, Reinfection, and Vaccine Effectiveness in Arizona Frontline and Essential Workers: Protocol for a Longitudinal Cohort Study

**DOI:** 10.2196/28925

**Published:** 2021-06-24

**Authors:** Karen Lutrick, Katherine D Ellingson, Zoe Baccam, Patrick Rivers, Shawn Beitel, Joel Parker, James Hollister, Xiaoxiao Sun, Joe K Gerald, Kenneth Komatsu, Elizabeth Kim, Bonnie LaFleur, Lauren Grant, Young M Yoo, Archana Kumar, Julie Mayo Lamberte, Benjamin J Cowling, Sarah Cobey, Natalie J Thornburg, Jennifer K Meece, Preeta Kutty, Janko Nikolich-Zugich, Mark G Thompson, Jefferey L Burgess

**Affiliations:** 1 Family and Community Medicine College of Medicine - Tucson University of Arizona Tucson, AZ United States; 2 Epidemiology and Biostatistics Department Mel and Enid Zuckerman College of Public Health University of Arizona Tucson, AZ United States; 3 Mel and Enid Zuckerman College of Public Health University of Arizona Tucson, AZ United States; 4 Department of Community, Environment and Policy Mel and Enid Zuckerman College of Public Health University of Arizona Tucson, AZ United States; 5 Arizona Department of Health Services Phoenix, AZ United States; 6 BIO5 Institute University of Arizona Tucson, AZ United States; 7 Centers for Disease Control and Prevention Atlanta, GA United States; 8 School of Public Health University of Hong Kong Hong Kong China; 9 Department of Ecology and Evolution University of Chicago Chicago, IL United States; 10 Marshfield Clinic Research Institute Marshfield, WI United States; 11 Department of Immunobiology College of Medicine - Tucson University of Arizona Tucson, AZ United States

**Keywords:** SARS-CoV-2, COVID-19, health care personnel, first responders, essential workers

## Abstract

**Background:**

COVID-19 has spread worldwide since late 2019, with an unprecedented case count and death toll globally. Health care personnel (HCP), first responders, and other essential and frontline workers (OEWs) are at increased risk of SARS-CoV-2 infection because of frequent close contact with others.

**Objective:**

The Arizona Healthcare, Emergency Response, and Other Essential Workers Study (AZ HEROES) aims to examine the epidemiology of SARS-CoV-2 infection and COVID-19 illness among adults with high occupational exposure risk. Study objectives include estimating the incidence of SARS-CoV-2 infection in essential workers by symptom presentation and demographic factors, determining independent effects of occupational and community exposures on incidence of SARS-CoV-2 infection, establishing molecular and immunologic characteristics of SARS-CoV-2 infection in essential workers, describing the duration and patterns of real-time reverse transcription–polymerase chain reaction (rRT-PCR) positivity, and examining postvaccine immunologic response.

**Methods:**

Eligible participants include Arizona residents aged 18 to 85 years who work at least 20 hours per week in an occupation involving regular direct contact (ie, within 3 feet) with others. Recruitment goals are stratified by demographic characteristics (50% aged 40 years or older, 50% women, and 50% Hispanic or American Indian), by occupation (40% HCP, 30% first responders, and 30% OEWs), and by prior SARS-CoV-2 infection (with up to 50% seropositive at baseline). Information on sociodemographics, health and medical history, vaccination status, exposures to individuals with suspected or confirmed SARS-CoV-2 infection, use of personal protective equipment, and perceived risks are collected at enrollment and updated through quarterly surveys. Every week, participants complete active surveillance for COVID-like illness (CLI) and self-collect nasal swabs. Additional self-collected nasal swab and saliva specimens are collected in the event of CLI onset. Respiratory specimens are sent to Marshfield Laboratories and tested for SARS-CoV-2 by rRT-PCR assay. CLI symptoms and impact on work and productivity are followed through illness resolution. Serum specimens are collected every 3 months and additional sera are collected following incident rRT-PCR positivity and after each COVID-19 vaccine dose. Incidence of SARS-CoV-2 infections will be calculated by person-weeks at risk and compared by occupation and demographic characteristics as well as by seropositivity status and infection and vaccination history.

**Results:**

The AZ HEROES study was funded by the US Centers for Disease Control and Prevention. Enrollment began on July 27, 2020; as of May 1, 2021, a total of 3165 participants have been enrolled in the study. Enrollment is expected to continue through December 1, 2021, with data collection continuing through at least April 2022, contingent upon funding.

**Conclusions:**

AZ HEROES is unique in aiming to recruit a diverse sample of essential workers and to prospectively follow strata of SARS-CoV-2 seronegative and seropositive adults. Survey results combined with active surveillance data on exposure, CLI, weekly molecular diagnostic testing, and periodic serology will be used to estimate the incidence of symptomatic and asymptomatic SARS-CoV-2 infection, assess the intensity and durability of immune responses to natural infection and COVID-19 vaccination, and contribute to the evaluation of COVID-19 vaccine effectiveness.

**International Registered Report Identifier (IRRID):**

DERR1-10.2196/28925

## Introduction

COVID-19, caused by the betacoronavirus SARS-CoV-2, has spread worldwide since late 2019, with at least 80 million confirmed cases and 1.8 million deaths reported globally in the year since discovery of the virus [1].

COVID-like illness (CLI) includes symptoms of fever or chills, cough, shortness of breath, sore throat, diarrhea, muscle aches, and loss of smell or taste [2]. Outbreak reports and mass testing initiatives suggest that a substantial proportion of individuals who test positive have no symptoms [3-6]. However, the proportion of confirmed cases with true asymptomatic infection is likely overestimated due to incomplete symptom assessment, inadequate follow-up to accurately classify presymptomatic individuals, and potential misclassification of those with previously unrecognized infection as asymptomatic [7]. Immigrant, racial and ethnic minority, and low-income communities have experienced a disproportionate burden of SARS-CoV-2 infections [8]. Severity of illness also differs by sociodemographic characteristics, with older adults and those with underlying health conditions at the highest risk of severe outcomes and death [9]. Optimizing public health responses requires further scientific study to estimate the incidence of infection, severity of illness, and variable immune response in sociodemographically diverse communities at high risk for infection [10].

Individuals in certain occupations are at increased risk of SARS-CoV-2 infection because of frequent close contact with others, including health care personnel (HCP), first responders, and other essential and frontline workers (OEWs). Recent studies highlight the elevated risk of exposure and infection among HCP [11-13], with more severe outcomes experienced by racial and ethnic minority HCP [14,15]. Less evidence exists, however, on transmission risks and severity of illness among OEWs [16,17]. First responders face a variety of exposures, including entering the homes of people with unknown disease status and performing aerosol-generating procedures, such as resuscitation [18]. Other essential and/or frontline occupational sectors with potentially high SARS-CoV-2 exposure include schools and childcare, agriculture and food production, energy, water and wastewater, retail (eg, grocery stores and warehouses), trades (eg, construction and plumbing), and nonprofits and social service organizations [19-21].

Prospective monitoring of symptomatic and asymptomatic infection is necessary to assess the combined epidemiological, immunological, and clinical impact of COVID-19 among essential workers [22]. Recent studies suggest that SARS-CoV-2 immunoglobulins M, G, and A (IgM, IgG, and IgA) are detected in various combinations in variable numbers of individuals in the first 2 weeks of infection, with IgG detected in most subjects after 14 days [23-25]. Although antibody response peaks at 16 to 42 days postinfection [23-26], and IgM and IgA levels decline, levels of IgG against the spike protein remain stable; protective immunity of at least 6 months has been measured, and much longer immunity is anticipated based on previous studies of coronaviruses—up to 17 years for SARS-CoV-1—and other acute viral infections [27-29]. Factors associated with variable immune response, including neutralizing (no infection), protective (mild to asymptomatic infection), or enhanced (severe symptoms) [30,31], are not yet fully understood for SARS-CoV-2.

The US Food and Drug Administration (FDA) has authorized the distribution of the first COVID-19 vaccines under Emergency Use Authorization (EUA), and the US Centers for Disease Control and Prevention (CDC) has recommended prioritization of vaccination for HCP, first responders, and OEW populations [32,33]. However, there is limited knowledge of vaccine intent and hesitancy among essential workers and of vaccine efficacy among those who were seropositive for SARS-CoV-2 prior to vaccination.

The Arizona Healthcare, Emergency Response, and Other Essential Workers Study (AZ HEROES) will follow a cohort of 4000 essential workers who are demographically representative of Arizona and stratified by prior exposure to SARS-CoV-2, such that about half are seronegative and half seropositive. The study affords researchers the opportunity to assess multiple knowledge gaps regarding the epidemiology and immunology of SARS-CoV-2 infections among a diverse statewide sample of essential workers with high exposure potential. AZ HEROES has also been expanded to evaluate immune parameters and incidence of SARS-CoV-2 infection following COVID-19 vaccination in previously seronegative and seropositive individuals (Table 1).

**Table 1:** Primary and secondary objectives for the AZ HEROES^a^ study of SARS-CoV-2 infection and immunity in a statewide cohort of essential workers stratified by seronegative and seropositive cohorts.

Type of objective	Objectives per cohort	
	Seronegative cohort^b^	Seropositive cohort^c^
Primary	Estimate incidence of SARS-CoV-2 infection in essential workers by symptom presentation and demographic factors Estimate the relative risk of infection with SARS-CoV-2 versus influenza during influenza season Determine independent effects of occupational and community exposures on incidence of SARS-CoV-2 infection	Establish molecular and immunologic characteristics of SARS-CoV-2 infection in essential workers Describe the duration and patterns of rRT-PCR^d^ positivity Describe levels of total antibodies, neutralizing antibodies, and other immune parameters over time Examine postvaccine immunologic response in those previously infected
Secondary	Examine the role of knowledge, attitudes, and practices related to SARS-CoV-2 in exposures and incident infection Identify predictors of vaccine hesitancy and uptake by occupation Compare incidence of infection in vaccinated vs unvaccinated essential workers	Describe severity and impact of illness on essential workers Examine predictors of severe disease among those naturally infected with SARS-CoV-2 Estimate incidence of SARS-CoV-2 reinfection Assess predictors of vaccine hesitancy and uptake among those with history of natural infection

^a^AZ HEROES: Arizona Healthcare, Emergency Response, and Other Essential Workers Study.

^b^Includes person-time contributed by individuals enrolled as seronegative who do not become naturally infected during the study *and* person-time contributed by individuals enrolled as seronegative *before* becoming infected during the study.

^c^Includes person-time contributed by individuals enrolled with prior evidence—viral or serologic—of SARS-CoV-2 infection *and* person-time contributed by individuals enrolled as seronegative *after* becoming naturally infected during the course of the study.

^d^rRT-PCR: real-time reverse transcription–polymerase chain reaction.

## Methods

### Study Design

**Figure 1:**
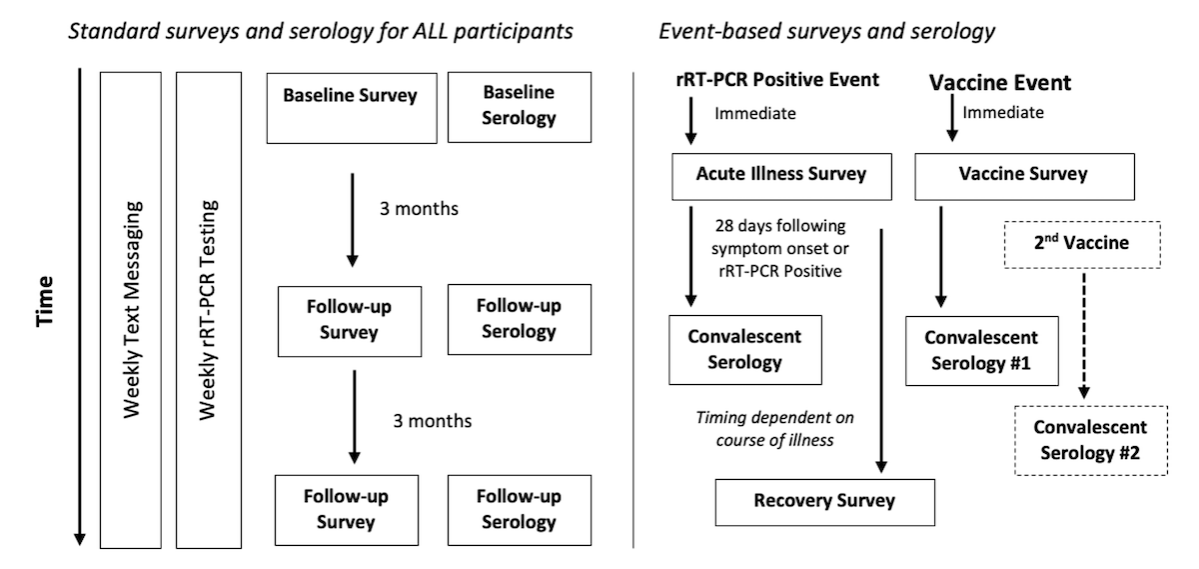
AZ HEROES flow of activities for baseline, follow-up, and event-based surveys and serology occurring in the context of weekly rRT-PCR testing and text-based symptom surveillance. AZ HEROES: Arizona Healthcare, Emergency Response, and Other Essential Workers Study; rRT-PCR: real-time reverse transcription–polymerase chain reaction.

AZ HEROES is a prospective cohort study with the goal of following 4000 essential workers, including 2000 seronegative and 2000 seropositive—defined as evidence of SARS-CoV-2 infection by serologic testing—individuals throughout the state of Arizona (Figure 1). The study duration is planned for July 2020 through April 2022.

### Setting

The state of Arizona has 7.2 million people residing in 15 counties and 22 sovereign American Indian communities. Nine of Arizona’s counties are designated as rural [34], although the majority of the population resides in the greater Phoenix and Tucson metropolitan areas. Arizona has large minority populations, with 32% Hispanic and 5% American Indian populations [35].

### Participants

#### Eligibility Criteria

Eligible participants include Arizona residents aged 18 to 85 years who currently work at least 20 hours per week in an occupation involving regular direct contact (ie, within 3 feet) with others, assessed at the participant level. We have intentionally chosen a broad occupational category for frontline and essential workers in order to capture the full breadth of occupations that cannot socially distance when conducting their work [36], as well as an inclusive age range because 13.9% of the Arizona workforce is over the age of 65 years [37]. The occupations are categorized as HCP, first responders, or OEWs. HCP include clinical providers and support staff in inpatient, outpatient, or residential settings. First responders include firefighters, emergency medical services, law enforcement, border patrol, and correctional officers. OEWs include workers in the following sectors: education, agriculture and food processing, public and other transportation services, solid waste collection, warehouse and delivery, utilities, government and community-based services, childcare, information technology, environmental services, and hospitality. All participants must have access to a smartphone or internet-connected computer, a mailing address, and the ability to speak or write English or Spanish. Exclusion criteria include receipt of a COVID-19 vaccine prior to enrollment, although we continue to follow participants who are vaccinated during the study. The majority of the cohort of the HCP and first responders were recruited prior to vaccine availability.

#### Recruitment Strategy

In order to enroll 4000 participants as quickly as possible, we have employed a multipronged recruitment strategy. First, we are recruiting from ongoing Arizona-based COVID-19 testing activities, such as university-driven antibody and saliva testing initiatives and serology surveillance conducted by the state health department in selected occupations (eg, nursing homes and correctional facilities). Second, we have partnered with community-based COVID-19 cohorts to recruit participants. The selected cohorts are low-touch and send periodic surveys to participants with little to no overlap in scope. For the testing and existing cohort populations, we directly contact individuals that indicate they would be willing to be contacted for future research. Third, the study accepts self-referrals, so we have developed a marketing strategy to increase general study awareness through press releases, targeted recruitment to occupations, and social media.

All recruitment and enrollment activities are conducted remotely utilizing a virtual call-center platform and REDCap (Research Electronic Data Capture) [38] to ensure staff and participant safety. Direct recruitment is conducted via phone and email. Participants are given the option to complete a self-screening questionnaire survey that is emailed to them or to complete a screening interview over the phone. Once deemed eligible and the participant is interested in the study, an electronic consent form is emailed to participants to review and sign electronically through REDCap.

**Figure 2:**
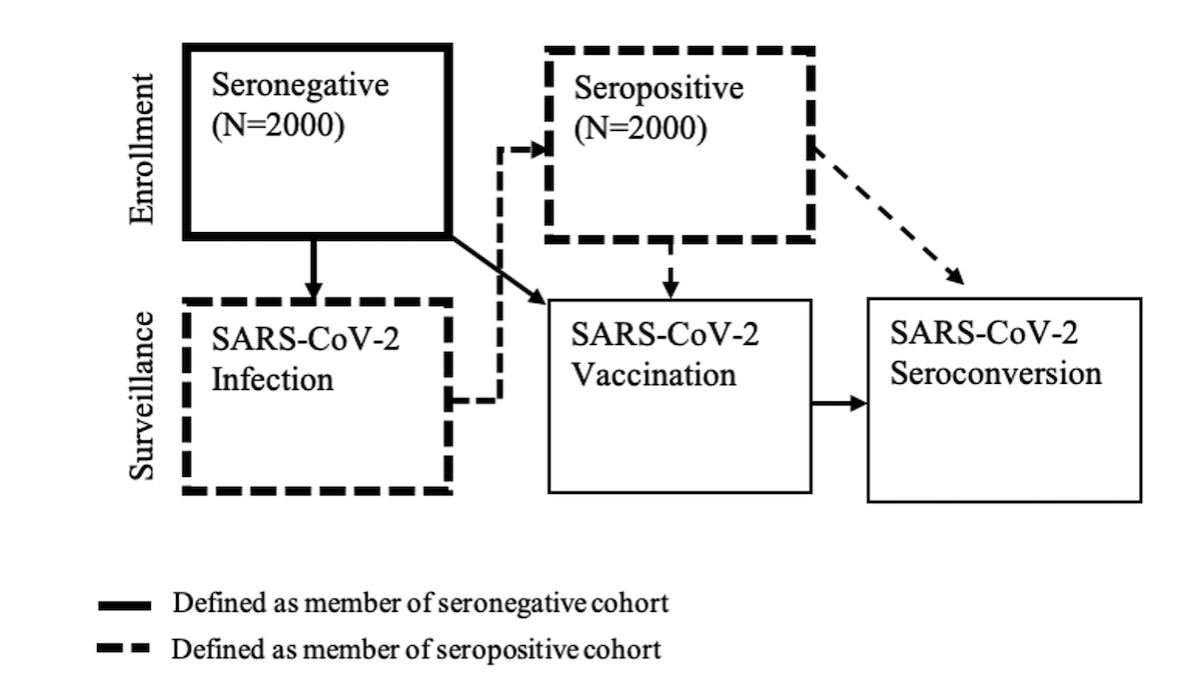
Participants who enroll as seronegative will switch to the seropositive cohort during the course of the study if they become infected as confirmed by rRT-PCR or serology prior to vaccination. rRT-PCR: real-time reverse transcription–polymerase chain reaction.

Sampling targets are based on the employment demographics of Arizona, and we seek to enroll essential workers in the following proportions: 50% from 18 to 40 years old and 50% between 41 and 85 years old, 50% women, and 50% Hispanic or American Indian. By occupation, we seek to enroll 40% HCP, 30% first responders, and 30% OEWs. These sampling breakdowns are presented in Table 2. Our goal is to enroll these proportions in both seronegative and seropositive specimens (Table 2). As specified targets are met, recruitment and enrollment priorities will shift to underenrolled groups and to replace individuals that become COVID-19 positive throughout the study (Figure 2).

**Table 2:** Enrollment strata for age, race, ethnicity, and occupation with minimum enrollment targets for participants in the AZ HEROES^a^ study.

Age group and race or ethnicity (target %)		Sex (target %)	Health care personnel (n=1600), n	First responders (n=1200), n	Other frontline and essential workers^b^ (n=1200), n
**18 to 39 years (50%)**					
	White and Non-Hispanic (50%)	Female (50%)	200	152	152
		Male (50%)	200	152	152
	Hispanic or Native American (50%)	Female (50%)	200	152	152
		Male (50%)	200	152	152
**40 to 85 years (50%)**					
	White and Non-Hispanic (50%)	Female (50%)	200	152	152
		Male (50%)	200	152	152
	Hispanic or Native American (50%)	Female (50%)	200	152	152
		Male (50%)	200	152	152

^a^AZ HEROES: Arizona Healthcare, Emergency Response, and Other Essential Workers Study.

^b^Includes frontline personnel who interact with the public as well as personnel who work in close proximity to each other (eg, call centers, warehouses, agriculture, and food processing).

#### Enrollment

Upon enrollment, participants are asked to complete a baseline questionnaire that collects information about sociodemographic characteristics; health status and behaviors; occupational exposure, tailored to the occupational category; history with and attitudes toward COVID-19; and influenza vaccination history during 2020-2021 and the previous five seasons (Table 3). Participants are asked to schedule a blood draw (40 mL) within 5 days of enrollment at a laboratory facility in their area in order to complete their baseline serology; a box of self-collection respiratory supplies are shipped to them so they can begin their active surveillance.

**Table 3:** Information sources and timing of collection for key variables through surveys, the bidirectional text platform, and specimen collection.

Collected information		Surveys				Active surveillance texts			Specimen submission		
		Screen	Baseline	Follow-up	Event based^a^	Weekly	Monthly (rotating)	Off schedule	Weekly	Quarterly	Event based^a^
**Demographics**											
	Age and gender	✓^b^									
	Race or ethnicity	✓									
	Date of birth	✓									
	Education and income		✓								
	Household composition		✓	✓							
	Occupation information	✓	✓	✓							
**Health and medical history**											
	Overall health		✓	✓		✓					
	Sleep quality		✓			✓					
	Tobacco history		✓								
	Comorbidities and medications		✓								
	Height and weight		✓								
	Pregnancy		✓	✓							
	Influenza infection								✓		
	Vaccine knowledge and attitudes		✓	✓			✓				
**SARS-CoV-2 infection**											
	rRT-PCR^c^ pre-enrollment	✓									
	rRT-PCR postenrollment								✓		✓
	Serology pre-enrollment	✓									
	Serology postenrollment									✓	✓
	Occupational exposure		✓	✓			✓				
	Community exposure		✓	✓			✓				
	Symptoms	✓			✓	✓		✓	✓		✓
	Illness duration and recovery			✓	✓						
	Illness severity and impact			✓	✓						
**Vaccination**											
	Influenza, history		✓								
	Influenza, current			✓							
	SARS-CoV-2, intent			✓				✓			
	SARS-CoV-2, 2020-2021				✓			✓			

^a^Events include acute illness, recovery, and vaccination, which prompt additional study follow-up.

^b^Checkmarks indicate the information was collected at the indicated time and by the indicated method.

^c^rRT-PCR: real-time reverse transcription–polymerase chain reaction.

### Active Surveillance

As part of active surveillance for incident SARS-CoV-2 infection, all participants provide weekly self-collected mid-turbinate nasal swabs appropriate to test for SARS-CoV-2 and influenza, during the influenza season. Upon enrollment, study participants are provided with information that the study duration could be up to 2 years, but initial expectations are for at least 36 weeks of weekly self-collected respiratory specimens. If an individual experiences CLI, they are asked to collect an additional respiratory specimen on the date of onset of the first CLI symptom. Weekly and illness kits are differentiated by color so participants know which to take and so study staff can track supplies. Respiratory specimens are analyzed utilizing the CDC-designated reference laboratory for real-time reverse transcription–polymerase chain reaction (rRT-PCR) assay testing. Specimens are tested and results obtained 2 to 5 days from collection. If participants receive a positive test result, trained study staff contact participants to provide CDC guidance on quarantine practices and warning signs requiring medical care as well as to answer any questions they may have.

AZ HEROES staff prepare and distribute self-collection kits to the study participants, including detailed paper and video instructions. The laboratory provides feedback on specimens that were unable to be tested because of participant error in collection or shipping of the sample (eg, leaking or missing required components). This feedback is utilized to re-educate participants.

Enrolled participants participate in active surveillance via weekly surveys, explained in detail in the Data Collection section.

### Data Collection

#### Overview

Active surveillance for acute illness is conducted throughout the study period. Participants are prompted to begin surveillance in the week following study enrollment and completion of the baseline survey. Each week, all participants are contacted via text message on their predesignated surveillance day (described in detail below). At the end of each text message exchange, the participant is reminded to collect a weekly specimen on their assigned day for collection.

#### Active Surveillance Surveys

As a part of active surveillance, participants are contacted weekly via secure SMS text messages, via Twilio, asking them two standardized questions about their general health status and presence of CLI symptoms. Twilio is a text messaging service that can read and write into the study REDCap database and customize questions based on participant responses. In addition to the two standardized questions, each week they receive one of four sets of rotating questions about changes in their occupational SARS-CoV-2 exposure, community and household exposure, and attitudes and beliefs surrounding COVID-19 risk. Any individual who indicates CLI in a weekly survey, or by contacting AZ HEROES staff directly, completes additional information via a mobile-friendly webform, including the participant’s symptoms, self-reported severity, duration, self-reported medical treatment, function during and after illness, and details about the resolution of their illness.

#### Self-reported Data

Participants who indicate they have experienced CLI in the last 7 days are moved to an acute illness monitoring flow, where they are instructed to collect and ship an acute illness kit and complete additional questions about their illness episode. Individuals can also be placed into the acute illness monitoring flow by notifying study staff that they are ill. Participants remain in the acute illness arm until they self-report that their illness has resolved. Before returning to the weekly active surveillance flow, participants complete a recovery survey in which they confirm duration of illness and answer questions about atypical symptoms, productivity loss, and use of health services. Participants continue to take weekly respiratory specimens throughout their acute illness monitoring.

#### Vaccine Information

Participants are asked a series of questions to assess their knowledge, attitudes, and practices (KAPs) related to SARS-CoV-2 vaccination in the enrollment and/or follow-up survey to capture the information prior to vaccination. Similar to previous KAP studies of influenza vaccines [39,40], participants are asked how much they know about the COVID-19 vaccines, if they received the vaccine, their intention to receive one if they have not, how safe and effective they think the vaccines are, and how likely they are to get sick if they do not receive a vaccine.

Through partnerships with state and county health departments, we track the timing and distribution of COVID-19 vaccines to know when they will be available to individuals within the study.

To capture vaccination information, participants are periodically asked if they have been vaccinated. If they have not, they indicate if they plan to be vaccinated in the upcoming 8 weeks, indicating how many weeks. The form is re-sent in 8 weeks if the participant indicates that they do not know when they will be vaccinated. Once vaccinated, participants complete a brief webform including date of vaccination, vaccine manufacturer, and order in sequence (eg, first or second) for vaccines requiring multiple doses. State Immunization Information System registries will be used as a backup to capture vaccine information about individuals who do not share the information with the study via text message, and for confirmation and completeness on individuals who do receive the vaccine. Participants consent to having their vaccination verified upon enrollment.

#### Laboratory Methods

##### Respiratory Specimens

Participants are asked to self-collect a respiratory specimen each week of the study period. Sampling kits are provided to all study participants, which include collection and shipping supplies for 8 weeks of collections, along with illustrated instructions on how to properly collect and ship their respiratory specimens. Study staff track the use of specimen kits and ship replenishments to participants as needed. Each week, regardless of symptoms, participants collect an anterior mid-turbinate nasal swab on both nares, using a flocked swab or equivalent, and place it into a tube containing viral transport media (VTM). If participants experience CLI, they use an *acute illness kit*, which consists of materials to collect a nasal swab in VTM and a saliva specimen in a saliva collection tube. All specimens are shipped with a cold pack, using priority overnight express shipping to a CDC-designated laboratory following International Air Transport Association guidelines [41]. Upon receipt by the laboratory, specimens are aliquoted and analyzed for SARS-CoV-2 using an rRT-PCR method [42] under FDA EUA. Remaining aliquots are maintained for additional analysis, banking, or long-term storage.

##### Blood Specimens

All participants contribute 40 mL of whole blood at enrollment, at 11- to 13-week intervals, and following positive rRT-PCR or vaccination events. Participants can submit specimens at participating laboratories closest to the participant’s residence or work. If a participant does not develop symptoms, but SARS-CoV-2 is detected in a weekly specimen, participants are instructed to submit a blood sample approximately 4 weeks following the date of first rRT-PCR detection; if the participant experiences CLI within 2 weeks of virus detection, they are instructed to submit a blood sample 4 weeks after initial symptom onset. If the participant has a convalescent blood specimen drawn prior to another planned repeat blood collection, the scheduling of the following blood collections will be 11 to 13 weeks following the convalescent draw. Participants who receive the COVID-19 vaccine during the study period are asked to provide a blood specimen 14 to 21 days after each dose of the vaccine—with the first postvaccination blood draw collected prior to the second vaccination dose, if relevant—and then every 11 to 13 weeks as described above. Information on adverse events and symptoms related to vaccination will be collected retrospectively after participants receive both doses of the vaccine.

Whole blood is collected and processed using CDC guidelines for serum collection [43]. The serum specimens are divided into aliquots labeled with the same study ID and specimen ID on all tubes, and an aliquot ID unique to each tube. All specimens are stored at −70 °C or colder prior to SARS-CoV-2 antibody analysis or long-term storage. At the University of Arizona, the serum is tested for antibodies against the receptor binding domain of the spike protein and verified with the S2 domain of S protein antibodies, as previously described [24], using the FDA EUA test (ID 201116). This testing at study entry is used to ensure correct placement of AZ HEROES participants into seronegative or seropositive groups.

#### Data Collection and Security

Most research activities occur through electronic communications (ie, email, text, and internet-based surveys), telephone contacts, or via postal or express mail, minimizing direct contact between study staff and participants. All surveys are self-administered by participants on a computer or smartphone. Surveys can also be administered by telephone or mail should participants be unable or become unwilling to access them online. Participant information given to study staff via phone or email conversation is entered and stored in the REDCap database by study staff. Alternatively, data are imported into the REDCap database from Twilio for participant responses to text surveillance or by direct participant response into the REDCap database.

#### Data Management

##### REDCap

A study database is maintained in REDCap. Tracking databases with patient identifiers and contact information are securely kept according to the University of Arizona standard operating procedures with respect to cybersecurity, privacy, patient confidentiality, and compliance with applicable patient privacy regulations. Any study-related documents with personal identifiers are stored in a locked cabinet in lockable offices on campus. All study-related documents and specimens contain a unique identiﬁer for each participant. Data entry forms provide some quality assurance using logic and range checks as well as automated skip patterns. The research team performs additional data quality checks on a weekly basis, including assessments of missing data. Laboratory results are entered directly into the REDCap study database from the study reference laboratory, including results from rRT-PCR assays and serologic assays. If a reference laboratory is not able to enter data directly, the laboratory is provided with a laboratory results reporting template that is then merged with study data using the specimen ID.

##### Twilio

Twilio is a cloud-based communications platform that allows for automated text messaging chains to be sent to study participants. It is used to send weekly and illness monitoring questions to participants. Participant responses are stored in Twilio until sent as a batch to the REDCap database once per day.

#### Statistical Considerations

##### Power Analysis

Our goal is to recruit 4000 participants, split evenly between seronegative and seropositive individuals. Among the seronegative cohort, we estimated that a sample of more than 852 participants is required to achieve 80% power (α=.05) to detect a true incidence of SARS-CoV-2 infection of 4%; the enrolled cohort exceeds this sample estimate at the drafting of this report. Thus, we expect to be sufficiently powered to make overall estimates and estimates by two-level strata, such as age, sex, or HCP versus others. Power estimation for COVID-19 vaccine effectiveness (VE) was performed using Monte Carlo simulation to generate survival time over 12 months based on varying vaccine coverage—with quarterly increases in two-dose vaccine coverage from 0% to 80% among HCP, 70% for first responders, and 30% for OEWs—and varying SARS-CoV-2 incidence rate, from 0.67% to 1.42% monthly attack rate, using the equations proposed by Austin and a Cox marginal model [44]. Based on 1000 simulations, with 2000 participants in the seronegative stratum, the study is estimated to have over 80% power to detect a true VE of 75%. If the data are pooled with similar studies using common methodologies to a total of 5000 subjects, the combined analysis is estimated to have 99% power to detect a true VE of 75% using the same assumptions.

##### Data Analysis

To estimate the incidence of SARS-CoV-2 infection and the corresponding 95% confidence intervals in essential workers, we will fit negative binomial regression models with person-time at risk as an offset. We will examine incidence of rRT-PCR–confirmed infections by occupation, symptom presentation, close contact exposure, and demographic variables. Logistic regression and negative binomial models will be used to estimate the risk of infection in different occupational groups. In the logistic regression model, we will include the log-transformed person-weeks as the offset. The model will then be adjusted by symptom presentation, demographic factors, study site, and health care utilization. The VE (1 − confirmed cases of COVID-19 illness per 1000 person-weeks among vaccinated essential workers ÷ confirmed cases of COVID-19 illness per 1000 person-weeks among unvaccinated essential workers × 100%) with 95% confidence interval will be estimated by a negative binomial regression model. The potential confounders, such as study site and previously seropositive status, will be included in the model. We will apply nonlinear mixed models to describe individual and group mean trajectories in neutralizing antibody titers over time. We will classify and identify subgroups of cases by self-reported clinical severity, health care utilization, occupational and community exposures, and duration of symptoms. These models will help elucidate the patterns of serologic immunity.

### Ethical Considerations

This study was reviewed and approved by the Arizona Department of Health and the University of Arizona Institutional Review Boards (IRBs) (see Code of Federal Regulations, Title 45, Part 46.114 [45]). The CDC and the Arizona Department of Health Services (ADHS) IRBs have reviewed the project. The ADHS IRB has approved the project and the CDC IRB deferred to the University of Arizona IRB. The College of Public Health at the University of Arizona houses all IRB and required study documentation. All participants complete informed consent electronically through the REDCap study database system. Research staff verify that participants understand key study activities, are aware of risks, and agree to participate prior to countersigning to confirm consent. Participants receive the results of their weekly and illness COVID-19 tests as well as the results of their antibody testing.

## Results

The AZ HEROES study was funded by the CDC. Enrollment began on July 27, 2020; as of May 1, 2021, a total of 3165 participants have been enrolled in the study. Enrollment is expected to continue through December 1, 2021, with data collection continuing through at least April 2022, contingent upon funding.

## Discussion

### Overview

Submission of weekly and CLI-onset swabs for SARS-CoV-2 rRT-PCR testing by AZ HEROES study participants is a high priority, as it allows researchers to estimate incidence of symptomatic and asymptomatic COVID-19. Currently, interest in receiving weekly nasal swab testing is high, with participants considering regular testing to be a substantial benefit, given high SARS-CoV-2 transmission rates in the community. When transmission rates in the community drop, or seropositivity increases due to natural infection or vaccination, this testing may be perceived by the study participants as more of a burden than a benefit. To maintain submission at acceptable rates at later time points in the study, monetary incentives have been included in the protocol. Methods for participant engagement through newsletters and reminders from study staff continue to evolve as the pandemic unfolds.

### Strengths

One strength of the study is the inclusion of 4000 individuals with substantial occupational exposure to SARS-CoV-2 owing to their work as HCP, first responders, and OEWs. Additionally, the enrolled population will be generally representative of the racial and ethnic demographics of Arizona, which ensures inclusion of high-risk groups, such as Hispanic and American Indian communities, previously found to be at increased risk for COVID-19 [8]. The longitudinal cohort study design allows for ongoing consistent and comprehensive symptom assessment, exposure assessment, and examination of KAPs related to SARS-CoV-2 infection and vaccination. Further, the serial blood sampling component of the study enables us to fully examine variations in immune response to infection and vaccination.

### Limitations

This study has several limitations. First, the ability to generalize trends related to infection incidence, disease severity, and immunologic response in our population of essential workers will likely be biased by the healthy worker effect. Second, the information retrieved from participants is principally self-reported or self-collected, which might introduce recall bias, particularly if participants do not complete rRT-PCR specimen collection and surveys consistently. Third, there may be a sampling bias related to the requirement that participants utilize computers and phones to complete surveys. Finally, not meeting enrollment targets and/or the length of the study period (1 year) may hamper the ability to assess reinfection rates, for example, because immunity for SARS-CoV-2 might last for more than 1 year.

### Conclusions

In conclusion, the design, recruitment, enrollment, and research activities of the AZ HEROES study provide a unique opportunity to improve our understanding of the incidence of SARS-CoV-2 infection, disease course, antibody and vaccine response, and KAPs among essential workers in the state of Arizona.

## Abbreviations

ADHS: Arizona Department of Health Services
AZ HEROES: Arizona Healthcare, Emergency Response, and Other Essential Workers Study
CDC: Centers for Disease Control and Prevention
CLI: COVID-like illness
EUA: Emergency Use Authorization
FDA: Food and Drug Administration
HCP: health care personnel
IgA: immunoglobulin A
IgG: immunoglobulin G
IgM: immunoglobulin M
IRB: Institutional Review Board
KAP: knowledge, attitude, and practice
OEW: other essential and frontline worker
REDCap: Research Electronic Data Capture
rRT-PCR: real-time reverse transcription–polymerase chain reaction
VE: vaccine effectiveness
VTM: viral transport media

## References

[1] (2020). COVID-19 dashboard by the Center for Systems Science and Engineering (CSSE). Johns Hopkins University Coronavirus Resource Center.

[2] (2020). Symptoms of COVID-19. Centers for Disease Control and Prevention.

[3] Costa S, Giavina-Bianchi P, Buss L, Mesquita Peres CH, Rafael MM, Dos Santos LGN, Bedin AA, Francisco MCPB, Satakie FM, Jesus Menezes MA, Dal Secco LM, Rodrigues Caron DM, de Oliveira AB, de Faria MFL, de Aurélio Penteado AS, de Souza IOM, de Fatima Pereira G, Pereira R, Matos Porto AP, Sanchez Espinoza EP, Mendes-Correa MC, Dos Santos Lazari C, Kalil J, de Moliterno Perondi MB, de Oliveira Bonfa ESD, Perreira AJ, Sabino E, da Silva Duarte AJ, Segurado AC, Dos Santos VA, Levin AS (2020). SARS-CoV-2 seroprevalence and risk factors among oligo/asymptomatic healthcare workers(HCW): Estimating the impact of community transmission. Clin Infect Dis.

[4] Kimball A, Hatfield KM, Arons M, James A, Taylor J, Spicer K, Bardossy AC, Oakley LP, Tanwar S, Chisty Z, Bell JM, Methner M, Harney J, Jacobs JR, Carlson CM, McLaughlin HP, Stone N, Clark S, Brostrom-Smith C, Page LC, Kay M, Lewis J, Russell D, Hiatt B, Gant J, Duchin JS, Clark TA, Honein MA, Reddy SC, Jernigan JA, CDC COVID-19 Investigation Team (2020). Asymptomatic and presymptomatic SARS-CoV-2 infections in residents of a long-term care skilled nursing facility - King County, Washington, March 2020. MMWR Morb Mortal Wkly Rep.

[5] Rivera F, Safdar N, Ledeboer N, Schaack G, Chen DJ, Munoz-Price LS (2020). Prevalence of SARS-CoV-2 asymptomatic infections in two large academic health systems in Wisconsin. Clin Infect Dis.

[6] Oran DP, Topol EJ (2020). Prevalence of asymptomatic SARS-CoV-2 infection. Ann Intern Med.

[7] Meyerowitz EA, Richterman A, Bogoch II, Low N, Cevik M (2021). Towards an accurate and systematic characterisation of persistently asymptomatic infection with SARS-CoV-2. Lancet Infect Dis.

[8] Strully K, Yang T, Liu H (2021). Regional variation in COVID-19 disparities: Connections with immigrant and Latinx communities in US counties. Ann Epidemiol.

[9] Yang Y, Peng F, Wang R, Yange M, Guan K, Jiang T, Xu G, Sun J, Chang C (2020). The deadly coronaviruses: The 2003 SARS pandemic and the 2020 novel coronavirus epidemic in China. J Autoimmun.

[10] Buitrago-Garcia D, Egli-Gany D, Counotte MJ, Hossmann S, Imeri H, Ipekci AM, Salanti G, Low N (2020). Occurrence and transmission potential of asymptomatic and presymptomatic SARS-CoV-2 infections: A living systematic review and meta-analysis. PLoS Med.

[11] Cheng VC, Wong S, Yuen K (2020). Estimating coronavirus disease 2019 infection risk in health care workers. JAMA Netw Open.

[12] Nguyen L, Drew DA, Graham MS, Joshi AD, Guo CG, Ma W, Mehta RS, Warner ET, Sikavi DR, Lo CH, Kwon S, Song M, Mucci LA, Stampfer MJ, Willett WC, Eliassen AH, Hart JE, Chavarro JE, Rich-Edwards JW, Davies R, Capdevila J, Lee KA, Lochlainn MN, Varsavsky T, Sudre CH, Cardoso MJ, Wolf J, Spector TD, Ourselin S, Steves CJ, Chan AT, COronavirus Pandemic Epidemiology Consortium (2020). Risk of COVID-19 among front-line health-care workers and the general community: A prospective cohort study. Lancet Public Health.

[13] Lynch J, Davitkov P, Anderson DJ, Bhimraj A, Cheng VCC, Guzman-Cottrill J, Dhindsa J, Duggal A, Jain MK, Lee GM, Liang SY, McGeer A, Lavergne V, Murad MH, Mustafa RA, Morgan RL, Falck-Ytter Y, Sultan S (2020). Infectious Diseases Society of America guidelines on infection prevention for health care personnel caring for patients with suspected or known COVID-19. Clin Infect Dis.

[14] Tai D, Shah A, Doubeni CA, Sia IG, Wieland ML (2021). The disproportionate impact of COVID-19 on racial and ethnic minorities in the United States. Clin Infect Dis.

[15] Moore JT, Ricaldi JN, Rose CE, Fuld J, Parise M, Kang GJ, Driscoll AK, Norris T, Wilson N, Rainisch G, Valverde E, Beresovsky V, Agnew Brune C, Oussayef NL, Rose DA, Adams LE, Awel S, Villanueva J, Meaney-Delman D, Honein MA, COVID-19 State‚ Tribal‚ Local‚ and Territorial Response Team (2020). Disparities in incidence of COVID-19 among underrepresented racial/ethnic groups in counties identified as hotspots during June 5-18, 2020 - 22 States, February-June 2020. MMWR Morb Mortal Wkly Rep.

[16] Rogers TN, Rogers CR, VanSant-Webb E, Gu LY, Yan B, Qeadan F (2020). Racial disparities in COVID-19 mortality among essential workers in the United States. World Med Health Policy.

[17] Rao A, Ma H, Moloney G, Kwong JC, Jüni P, Sander B, Kustra R, Baral SD, Mishra S A disproportionate epidemic: COVID-19 cases and deaths among essential workers in Toronto, Canada. medRxiv..

[18] Emergency response workers and employers. United States Department of Labor, Occupational Safety and Health Administration.

[19] (2020). Essential services and critical infrastructure. Centers for Disease Control and Prevention.

[20] Baker MG, Peckham TK, Seixas NS (2020). Estimating the burden of United States workers exposed to infection or disease: A key factor in containing risk of COVID-19 infection. PLoS One.

[21] (2020). Coronavirus disease (COVID-19): Health and safety in the workplace. World Health Organization.

[22] CDC COVID-19 Response Team (2020). Characteristics of health care personnel with COVID-19 - United States, February 12-April 9, 2020. MMWR Morb Mortal Wkly Rep.

[23] Long Q, Liu B, Deng H, Wu G, Deng K, Chen Y, Liao P, Qiu J, Lin Y, Cai X, Wang D, Hu Y, Ren J, Tang N, Xu Y, Yu L, Mo Z, Gong F, Zhang X, Tian W, Hu L, Zhang X, Xiang J, Du H, Liu H, Lang C, Luo X, Wu S, Cui X, Zhou Z, Zhu M, Wang J, Xue C, Li X, Wang L, Li Z, Wang K, Niu C, Yang Q, Tang X, Zhang Y, Liu X, Li J, Zhang D, Zhang F, Liu P, Yuan J, Li Q, Hu J, Chen J, Huang A (2020). Antibody responses to SARS-CoV-2 in patients with COVID-19. Nat Med.

[24] Ripperger T, Uhrlaub JL, Watanabe M, Wong R, Castaneda Y, Pizzato HA, Thompson MR, Bradshaw C, Weinkauf CC, Bime C, Erickson HL, Knox K, Bixby B, Parthasarathy S, Chaudhary S, Natt B, Cristan E, El Aini T, Rischard F, Campion J, Chopra M, Insel M, Sam A, Knepler JL, Capaldi AP, Spier CM, Dake MD, Edwards T, Kaplan ME, Scott SJ, Hypes C, Mosier J, Harris DT, LaFleur BJ, Sprissler R, Nikolich-Žugich J, Bhattacharya D (2020). Orthogonal SARS-CoV-2 serological assays enable surveillance of low-prevalence communities and reveal durable humoral immunity. Immunity.

[25] Iyer A, Jones FK, Nodoushani A, Kelly M, Becker M, Slater D, Mills R, Teng E, Kamruzzaman M, Garcia-Beltran WF, Astudillo M, Yang D, Miller TE, Oliver E, Fischinger S, Atyeo C, Iafrate AJ, Calderwood SB, Lauer SA, Yu J, Li Z, Feldman J, Hauser BM, Caradonna TM, Branda JA, Turbett SE, LaRocque RC, Mellon G, Barouch DH, Schmidt AG, Azman AS, Alter G, Ryan ET, Harris JB, Charles RC (2020). Persistence and decay of human antibody responses to the receptor binding domain of SARS-CoV-2 spike protein in COVID-19 patients. Sci Immunol.

[26] Lumley SF, O'Donnell D, Stoesser NE, Matthews PC, Howarth A, Hatch SB, Marsden BD, Cox S, James T, Warren F, Peck LJ, Ritter TG, de Toledo Z, Warren L, Axten D, Cornall RJ, Jones EY, Stuart DI, Screaton G, Ebner D, Hoosdally S, Chand M, Crook DW, O'Donnell A, Conlon CP, Pouwels KB, Walker AS, Peto TEA, Hopkins S, Walker TM, Jeffery K, Eyre DW, Oxford University Hospitals Staff Testing Group (2021). Antibody status and incidence of SARS-CoV-2 infection in health care workers. N Engl J Med.

[27] Baumgarth N, Nikolich-Žugich J, Lee FE, Bhattacharya D (2020). Antibody responses to SARS-CoV-2: Let's stick to known knowns. J Immunol.

[28] Callow KA, Parry HF, Sergeant M, Tyrrell DAJ (1990). The time course of the immune response to experimental coronavirus infection of man. Epidemiol Infect.

[29] Wu L, Wang N, Chang Y, Tian X, Na D, Zhang L, Zheng L, Lan T, Wang L, Liang G (2007). Duration of antibody responses after severe acute respiratory syndrome. Emerg Infect Dis.

[30] Coish JM, MacNeil AJ (2020). Out of the frying pan and into the fire? Due diligence warranted for ADE in COVID-19. Microbes Infect.

[31] To K, Hung IFN, Chan KH, Yuan S, To WK, Tsang DNC, Cheng VCC, Chen Z, Kok KH, Yuen KY (2021). Serum antibody profile of a patient with coronavirus disease 2019 reinfection. Clin Infect Dis.

[32] Advisory Committee on Immunization Practices (ACIP) (2020). COVID-19 ACIP vaccine recommendations. Centers for Disease Control and Prevention.

[33] McClung N, Chamberland M, Kinlaw K, Bowen Matthew D, Wallace M, Bell BP, Lee GM, Talbot HK, Romero JR, Oliver SE, Dooling K (2020). The Advisory Committee on Immunization Practices' ethical principles for allocating initial supplies of COVID-19 vaccine - United States, 2020. MMWR Morb Mortal Wkly Rep.

[34] (2020). Arizona - Rural definitions: State-level maps. Economic Research Service, US Department of Agriculture.

[35] QuickFacts: Arizona. United States Census Bureau.

[36] Roberts JD, Dickinson KL, Koebele E, Neuberger L, Banacos N, Blanch-Hartigan D, Welton-Mitchell C, Birkland TA (2020). Clinicians, cooks, and cashiers: Examining health equity and the COVID-19 risks to essential workers. Toxicol Ind Health.

[37] (2020). Local area unemployment statistics: Expanded state employment status demographic data. US Bureau of Labor Statistics.

[38] Harris PA, Taylor R, Minor BL, Elliott V, Fernandez M, O'Neal L, McLeod L, Delacqua G, Delacqua F, Kirby J, Duda SN, REDCap Consortium (2019). The REDCap consortium: Building an international community of software platform partners. J Biomed Inform.

[39] Wagner AL, Gordon A, Tallo VL, Simaku A, Porter RM, Edwards LJ, Duka E, Abu-Khader I, Gresh L, Sciuto C, Azziz-Baumgartner E, Bino S, Sanchez F, Kuan G, de Jesus JN, Simões EAF, Hunt DR, Arbaji AK, Thompson MG (2020). Intent to obtain pediatric influenza vaccine among mothers in four middle income countries. Vaccine.

[40] Hirsch A, Katz MA, Laufer Peretz A, Greenberg D, Wendlandt R, Shemer Avni Y, Newes-Adeyi G, Gofer I, Leventer-Roberts M, Davidovitch N, Rosenthal A, Gur-Arie R, Hertz T, Glatman-Freedman A, Monto AS, Azziz-Baumgartner E, Ferdinands JM, Martin ET, Malosh RE, Neyra Quijandría JM, Levine M, Campbell W, Balicer R, Thompson MG, SHIRI workgroup (2018). Study of Healthcare Personnel with Influenza and other Respiratory Viruses in Israel (SHIRI): Study protocol. BMC Infect Dis.

[41] The International Air Transport Association (2020). Dangerous Goods Regulations. 62nd edition.

[42] (2020). CDC’s diagnostic test for COVID-19 only and supplies. Centers for Disease Control and Prevention.

[43] (2020). Interim guidelines for collecting and handling of clinical specimens for COVID-19 testing. Centers for Disease Control and Prevention.

[44] Austin PC (2012). Generating survival times to simulate Cox proportional hazards models with time-varying covariates. Stat Med.

[45] (2009). Code of Federal Regulations. Title 45: Public Welfare, Part 46: Protection of Human Subjects.

